# Deep Learning for Infant Cry Recognition

**DOI:** 10.3390/ijerph19106311

**Published:** 2022-05-23

**Authors:** Yun-Chia Liang, Iven Wijaya, Ming-Tao Yang, Josue Rodolfo Cuevas Juarez, Hou-Tai Chang

**Affiliations:** 1 Department of Industrial Engineering and Management, Yuan Ze University, No. 135, Yuan-Tung Rd., Chung-Li Dist., Taoyuan City 32003, Taiwan; s1075445@mail.yzu.edu.tw (I.W.); josuercuevas@gmail.com (J.R.C.J.); houtai38@saturn.yzu.edu.tw (H.-T.C.); 2 Department of Chemical Engineering and Materials Science, Yuan Ze University, No. 135, Yuan-Tung Rd., Chung-Li Dist., Taoyuan City 32003, Taiwan; mingtao.yang.tw@gmail.com; 3 Far Eastern Memorial Hospital, No. 21, Sec. 2, Nanya S. Rd., Banciao Dist., New Taipei City 22000, Taiwan

**Keywords:** infant cry recognition, convolutional neuron network, long short-term memory, deep learning

## Abstract

Recognizing why an infant cries is challenging as babies cannot communicate verbally with others to express their wishes or needs. This leads to difficulties for parents in identifying the needs and the health of their infants. This study used deep learning (DL) algorithms such as the convolutional neural network (CNN) and long short-term memory (LSTM) to recognize infants’ necessities such as hunger/thirst, need for a diaper change, emotional needs (e.g., need for touch/holding), and pain caused by medical treatment (e.g., injection). The classical artificial neural network (ANN) was also used for comparison. The inputs of ANN, CNN, and LSTM were the features extracted from 1607 10 s audio recordings of infants using mel-frequency cepstral coefficients (MFCC). Results showed that CNN and LSTM both provided decent performance, around 95% in accuracy, precision, and recall, in differentiating healthy and sick infants. For recognizing infants’ specific needs, CNN reached up to 60% accuracy, outperforming LSTM and ANN in almost all measures. These results could be applied as indicators for future applications to help parents understand their infant’s condition and needs.

## 1. Introduction

Through language, humans deliver information to express their will. However, they have to learn from scratch. Lacking language, newborn babies are unable to express their specific desires. In general, a baby’s parents are its first teachers, and this interaction is the most crucial aspect of babies’ growth. Newborn babies express negative emotion or need by crying [1], often to the consternation of parents who cannot immediately ascertain the nature of this need.

Previous research has found that infants cry in fundamental frequencies that correlate to different factors, such as emotional state, health, gender, disease (abnormalities), pre-term vs. full-term, first cry, identity, etc. [2] In addition to these fundamental frequencies, infant cries have been subjected to signal analysis based on features including latency, duration, formant frequencies, pitch contour, and stop pattern [2].

Previous studies have sought to classify infant cries by type, with most focusing on using artificial intelligence approaches to predict physiological sensations such as hunger, pain, diaper change, and discomfort [3]. Some previous studies used pathological classes such as normal cries, hypo-acoustic (deaf) cries, and asphyxiating cries. For instance, Reyes–Galaviz and Arch–Tirado applied linear prediction and adaptive neuro-fuzzy inference system (ANFIS) analysis of the cry sound wave, successfully distinguishing fundamental frequencies among infants aged under 6 months [4].

Yong et al. used feature extraction to analyze infant cry signals, extracting 12 orders of mel-frequency cepstral coefficient (MFCC) features for model development [3]. Their developed model combined the convolutional neural network (CNN) and a stacked restricted Boltzmann machine (RBM). The model classified results as pathological based on the health status of the baby (sick vs. healthy), recognizing pathological conditions classified as hungry, in need of diaper changing, emotional needs, and in pain caused by medical treatment.

In using artificial intelligence approaches for building a classification model, feature selection plays a key role in determining model accuracy. On the other hand, deep learning approaches often provide satisfactory classification results, such as using artificial neural networks (ANN) or multi-layer perceptrons (MLP), CNN, and long-short term memory (LSTM); meanwhile, MFCC is commonly used for feature extraction in audio analysis. Therefore, this study sought to develop deep learning algorithms for infant cry classification.

The rest of this paper is organized as follows: Section 2 describes the methodology for data collection, data cleaning, feature extraction, and data analysis. The results are summarized and discussed in Section 3. The concluding remarks and future search are provided in Section 4.

## 2. Methodology

The cry signal was analyzed to extract important signal features [5]. One such feature was fundamental frequency in the range of 400 Hz to 500 Hz, compared with 200 Hz to 300 Hz for adults [6]. Other features for audio analysis include latency, duration, and formant frequency and are depicted as spectrograms for ease of use [2].

Data collection, data pre-processing, feature extraction, and data analysis will be detailed in the following subsections.

### 2.1. Data Collection

Audio data were collected by hospital nurses when the infants under their care began crying. The nurses would then note the condition they discovered to be the proximal cause of the infant crying:

- Hunger: The infant ceased crying when fed.
- Diaper change: The infant ceased crying following diaper change.
- Emotional needs: The infant ceased crying following physical touch/holding.
- Physiological Pain: Infant pain was caused by invasive medical treatment, including injection.

Infants were divided into “healthy” and “sick” datasets depending on whether they were in the nursery or neonatal intensive care unit (NICU), respectively. Data were recorded at the Far Eastern Memorial Hospital with infants in the nursery deemed healthy, while those in the ICU were deemed unhealthy. Anonymized audio signal recordings of infants (10 healthy infants aged 2 to 27 days in the nursery, and 6 neonatal ICU infants aged up to 4 months) were collected by an app and labeled by nursing staff at the Far Eastern Memorial Hospital, with IRB approval and informed parental consent.

Each audio recording had a duration of 10 s. Crying incidents lasting less than 10 s were not recorded. The app also determined that if an infant cried longer than 10 s, only the first 10 s of data was collected. There was then a 1 min resting time for the app to process and upload the data to the cloud. Finally, if an infant’s cry lasted more than 1 min and 10 s, the data were recorded as two separate cries.

### 2.2. Data Pre-Processing

Audio data quality highly depends on signal pre-processing [7,8]. Signal pre-processing eliminates irrelevant or unwanted information like noise and channel distortion [5].

The raw audio data collected from the hospital included noise which needed to be removed before modeling. Prior to cleaning, the data were retrieved from cloud storage in Wavpack format (.wav). The original 10 s audio clips were split into five 2 s clips and converted to 16-bit.wav files with a sampling rate of 8000 Hz.

One of the most important indicators of data cleaning performance is the removal of all bad data. Data were considered unclean data if 60% of the data matched these criteria: sound of adult human detected, data mislabeled, electronic/mechanical noise detected, sound of other infants detected, silence, etc.

### 2.3. Feature Extraction

**Figure 1:**
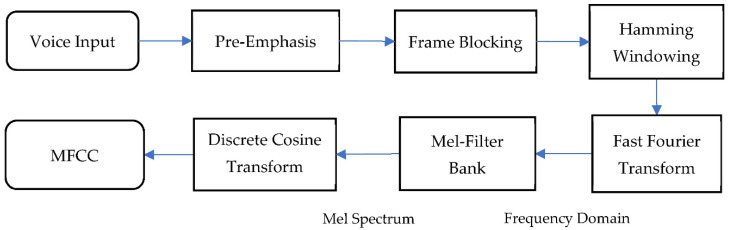
Block diagram of MFCC.

MFCC is widely used for feature extraction in audio analysis because of its highly efficient computer schemes and its robustness in distinguishing different noises [9]. MFCC effectively detects the human ear’s critical bandwidth frequencies used to retrieve important speech characteristics, and its procedure is shown in Figure 1.

In the first step, the audio was passed through a filter that emphasized higher frequencies. As a result of the loss of information in the higher frequencies, pre-emphasis was required to maintain the information in the higher frequencies.

The second step, framing, involved dividing audio signals into smaller segments. Framing was needed to get stationary information from part of the signal. In general, the width of the frame was around 20–30 ms. During this step, in order to prevent the adjacent frames from changing excessively, there was an overlap area between the two frames. The standard windows for MFCC were 25 ms frame with 10 ms overlap [10].

The next step was Hamming windowing, where each frame was multiplied in the hamming window. This step helped to reduce discontinuity in a signal by minimizing the spectral distortion at the beginning and the end of each frame. With Hamming window added to the spectrum, the intensity of the noise was reduced, and the peak representing the target signal was more apparent.

In fast Fourier transform (FFT), the frames were converted from the time domain to the frequency domain. The multiple-magnitude frequency passed through a triangular bandpass filter used to smooth the magnitude spectrum and to reduce the size of features involved.

From the frequency spectrum to a Mel spectrum, the Mel filter bank was the primary conversion step. The Mel-scale frequencies were distributed linearly in the low range, but logarithmically in the high range. Human ears are capable of hearing tones at frequencies lower than 1000 Hz on the linear scale [11]. The following equation was used to calculate this Mel filter bank:

(1) $$f_{Mel}=2595\times \log_{10}\left(1+\frac{freq}{700}\right)$$

Next, the log Mel spectrum was transformed using the discrete cosine transform (DCT). These features are similar to the spectrums and are commonly called Mel-scale cepstral coefficients. The Mel-scale cepstral coefficients were obtained from a frame of audio derived from the output of the DCT transforms of the MFCC.

### 2.4. Classification Model

The data are split into training, validation, and testing sets with a ratio of 70/15/15. The training data are used to train the model with backpropagation in each epoch to decrease the loss/error rate resulting from changing the weights used in the training process. Testing validated the robustness of the training process. Data used in the testing process were excluded from use in training.

#### 2.4.1. Artificial Neural Network (ANN)

**Figure 2:**
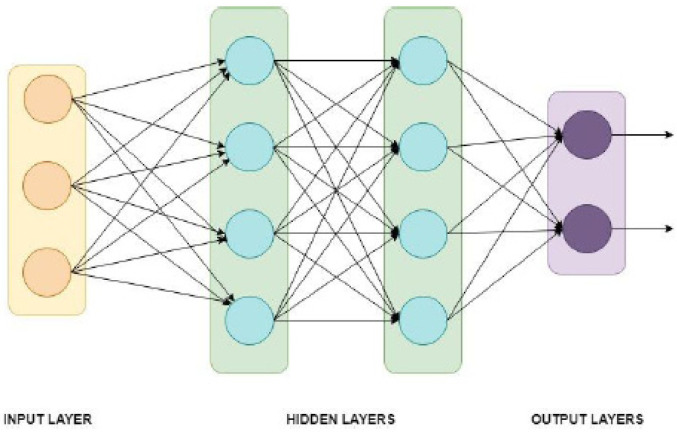
Artificial neural network structure for two-class classification [12].

Artificial neural network is used to find patterns in complex classification problems [12]. ANN is a machine learning algorithm using a dense layer as the perceptron. The ready-to-use MFCC was the input of the neural network, with 201 sequences for each coefficient and 12 × 201 sequences = 2412 values from each data as the input for the ANN layer. Figure 2 illustrates an ANN with three input neurons, two hidden layers (each with four hidden neurons), and two classes for the output.

#### 2.4.2. Convolutional Neural Network (CNN)

**Figure 3:**
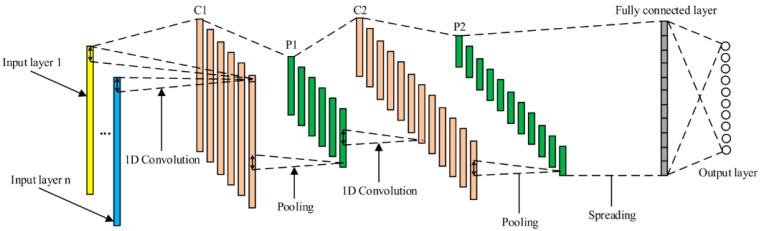
An illustrative example of the one-dimensional CNN structure [14].

Convolutional neural network is a neural network that contains many layers connecting all feature maps, allowing it to learn by its weights. Each layer can become the features layer [13,14]. CNN is widely used in image classification and audio processing due to its results and performance reliability. Figure 3 shows an illustrative example of a one-dimensional CNN structure.

The convolutional neural network retrieved feature maps for each layer, and the stride, padding, and kernel size were set in this layer. Kernel weights were learned during the training process in each neuron. Each position was multiplied by time series plus a bias term. Stride determined the step size of moving the kernel, while padding controlled the output result of the activation map. Several activation maps such as Sigmoid, ReLU, and hyperbolic tangent (tanh) were applied.

The pooling layer retrieved the output from the convolutional base on stride and padding that were set before. Two kinds of pooling were set: the maximum and the average of the result. Finally, the fully connected layer converted the last layer’s output and became the one-dimension result. The output took the biggest probability for the classification by using SoftMax function.

#### 2.4.3. Long Short-Term Memory (LSTM)

**Figure 4:**
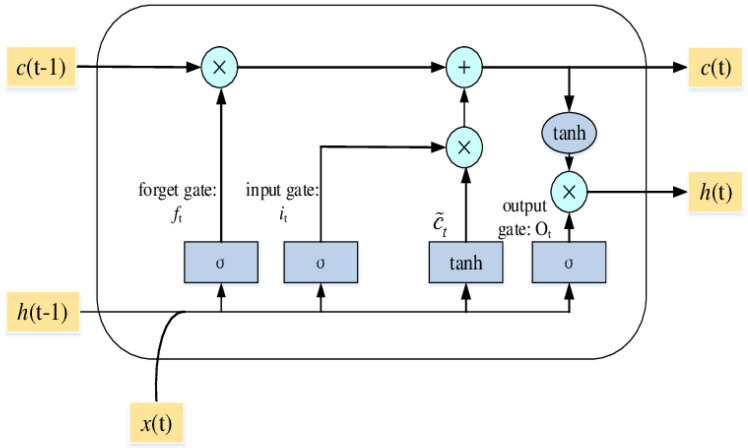
An illustrative example of the LSTM structure [14].

Long short-term memory is a method which avoids the vanishing gradient problem by which a neural network never reaches its optimal weight. This problem is caused by the error value disappearing during the backward process [15]. LSTM features three gates (input, forget, and output) that store important information, along with a one-cell state as illustrated in Figure 4.

As shown in Figure 4, the key step of LSTM is the cell state of *c*(t − 1). The horizontal line runs through the top of the diagram, like a conveyor belt straight down through the entire chain. The first step in LSTM is in the forget gate (*f_t_*). *h*(t − 1) and *x*(t) are numbers between 0 and 1 for each number in the cell state *c*(t − 1). The forget gate decision is made in the sigmoid function.

The next step is the input gate (*i_t_*), where the new information is either added in the cell state or is not added. Next is $\tilde{C}$*_t_*, where the new candidate could be added or not. This step determines the new input and either adds a new subject or replaces the old one.

Furthermore, the cell state needs to update *c*(t − 1) into the new cell state *c*(t). The old state *c*(t − 1) is multiplied by the old state *f_t_* which was decided to be forgotten earlier, and it is added to the product between *i_t_* and $\tilde{C}$*_t_*. These new values are scaled to decide how to update each state value. The last step is to get the output of the cell by using the tanh function (value between –1 and 1).

### 2.5. Classification of Experiments

This research consisted of several experiments. Depending on the number of classes considered, two-class, three-class, and four-class were investigated. The four-class consisted of the labels of the hungry class, diaper change class, emotional need class, and medical treatment class. The three-class removed the medical treatment class due to the relatively small number of data points. The two-class classification was to distinguish the health condition of the infant, i.e., healthy or sick. The full (unbalanced) dataset considered all the data samples, while the balanced dataset employed the down-sampling technique based on the smallest number of data points in any class considered. For example, since the medical treatment class owned only 88 data points, which was the smallest category, when considering the four-class classification, other three classes randomly selected 88 data points for further analysis.

## 3. Results and Discussions

### 3.1. Data Summary

**Figure 5:**
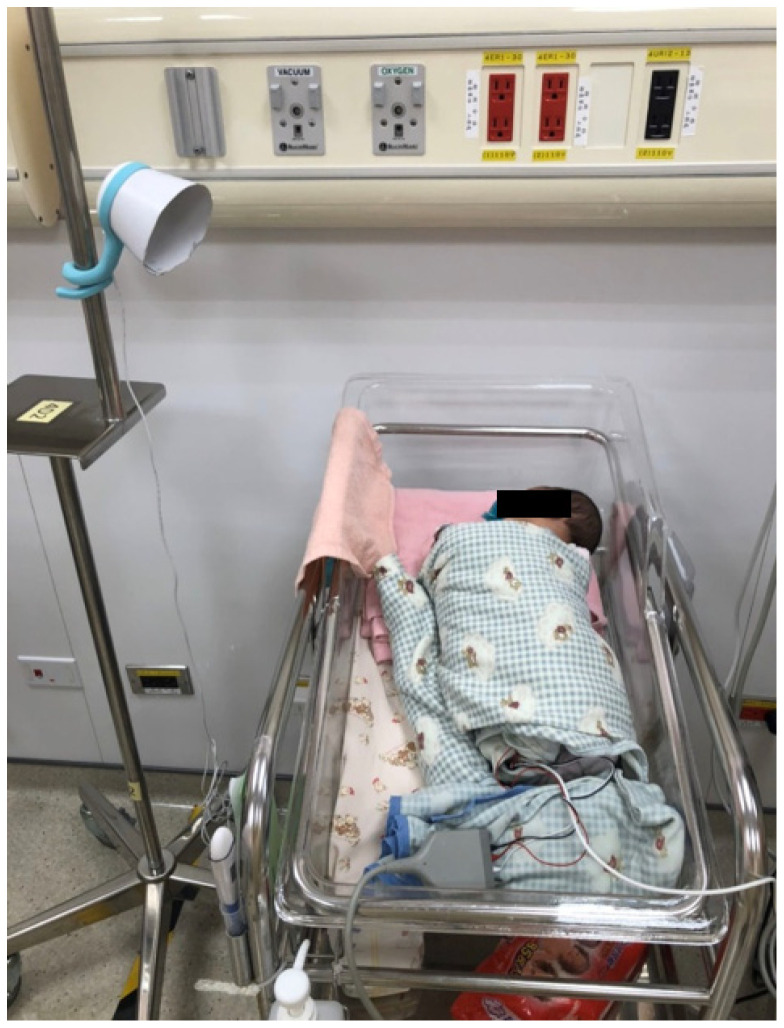
An example of the data collection device and the sample infant.

The infant cry data were collected from 33 babies in the neonatal intensive care unit and 26 babies from the nursery unit between October 2019 and January 2020 at the Far Eastern Hospital Memorial Hospital. A Lollipop baby monitor provided by Masterwork Aoitek Tech Corp was used in this study as the data collection tool. Figure 5 illustrates that each baby recorded in each unit was separate from the other babies in the unit and that the device was placed approximately 30 cm from the bed.

This Lollipop device was activated by the sounds of crying infants. Each sound was recorded for a period of ten seconds. If the cry lasted longer than ten seconds, the first ten seconds would be saved. There was then a one-minute rest period for the app to process and upload to the cloud. Additionally, if the baby cried for fewer than ten seconds, the app did not record their cry. Lastly, if a baby cried for more than one minute and ten seconds, two samples were collected consecutively. In collaboration with the nurses, an application embedded within a mobile device was used to label each recording. For the purposes of analysis, each recording was divided into five segments (each lasting two seconds).

After cleaning the data, the infants’ needs recognition was split into four classes. Table 1 shows the summary of the four-class dataset. The number of data points obtained from the healthy group was 1705, and the number of data points in the sick group were 840, nearly half of the healthy group. Also, the hungry class owned the most data points, i.e., 1171, and medical treatment was the least with only 88 samples. In order to limit the influence of unbalanced data, some balanced datasets were also established based on the number of samples in the smallest class (e.g., the medical treatment in the four-class, the diaper change in the three-class, and the sick group in the two-class, respectively). The data were randomly split into three categories: 70% for training, 15% for validation, and 15% for testing.

### 3.2. Parameter Setting

Parameter setting was divided into two parts: one for the MFCC and another for the classification methods. Table 2 provides the parameter values for the MFCC. In every MFCC, there were 201 sequences for each coefficient. All 12 coefficients were included in the consideration. Thus, there were 2412 values created (201 × 12 = 2412). This value became the input of the model and the dimension based on the model used.

Through the preliminary analysis, the parameter setting of each classification method—ANN, CNN, and LSTM—were summarized in Table 3. The five-fold cross-validation was employed for evaluating the performance of the proposed methods.

### 3.3. Experimental Results

Table 4, Table 5 and Table 6 show the precision and recall of the four-class, three-class, and two-class classification results, respectively. Table 4 and Table 5 show clear differences between the balanced and the full (imbalanced) datasets. The classes with the fewest data points in the full dataset, i.e., medical treatment in Table 4 and change diaper in Table 5, failed the prediction. However, the balanced data strategy showed tremendous improvement for precision and recall in changing diapers and medical treatment classes. For example, medical treatment’s precision improved from 7% to 53%, and the recall of change diapers improved from 24% to 53% in CNN in Table 4. Similar improvement can also be observed in the other two methods in Table 4 and Table 5. CNN performed the best of the three competing methods, while ANN showed inferior results. For the balanced dataset, CNN’s precision and recall ranged from 46% to 60% in the four-class and 55% to 61% in the three-class analyses.

As shown in Table 6, CNN and LSTM showed competitive performance in the two-class. CNN obtained 97% of healthy class’s precision and 98% of sick class’s recall in the balanced dataset, while LSTM had similar digits in those measures. Not surprisingly, ANN’s most inferior performance showed in the two-class case again. In addition, the balanced dataset also improved the performance of classification of all three methods in Table 6, although the gap was not as significant as the ones in Table 4 and Table 5.

Finally, the average accuracy over all classes is summarized in Table 7. Again, consistent with the precision and recall performance, CNN outperformed LSTM and ANN, and the balanced dataset helped improve the accuracy. For example, CNN’s average accuracy reached 64% and 60% in the four-class and three-class, respectively, while 96% of accuracy was obtained for the two-class.

The proposed methods could all distinguish the cry of an infant in healthy condition with high accuracy, precision, and recall. However, when it came to classifying psychological or physiological needs, the performance of classification deteriorated. This condition could be attributed to the dataset’s labeling errors, resulting in erroneous class predictions. For example, an infant may be in distress because it simultaneously is hungry and wants to be held. This kind of compound behavior is difficult to predict and is tough to be labeled by nurses.

## 4. Conclusions

The proposed deep learning approaches, CNN and LSTM, provided reliable and robust results for classifying sick and healthy infants based on recordings of infant cries. Recognition accuracy was improved by using a balanced dataset, with testing results of up to 64% on CNN for the four-class categorization. Better results were obtained in the health needs (two-class) test, possibly because of the data collection method employed, wherein the healthy and sick infants were diagnosed by doctors and were kept in two different rooms. This resulted in more controlled and accurate situations for data collection, as opposed to the emotional-state data collection, which presented the increased chance of mislabeling. Another possible reason for mislabeling was that an infant may have simultaneously experienced multiple stimuli resulting in crying behavior, making it difficult to isolate the actual proximal cause.

This study involved data samples with some unique characteristics such as race, age, residence area, and health status, as compared with other similar studies in the literature. Moreover, good data always play a major part in recognition performance. Improving the quality of data points is one way to get better recognition. Future work should seek to further improve data quality by better controlling the data collection environment, and additional feature extraction methods should be used for performance comparison against the MFCC feature set used here. The current dataset could also be combined with data from other hospitals, and dataset with age considerations is another way to boost the robustness of the model. In addition, the current model only included audio signals, and future work could integrate video signals to improve model robustness. Moreover, ensemble learning may offer performance improvements on the algorithmic side. Research involving data pertaining to multiple labels and experiments on different feature-extraction techniques can also be interesting areas for future investigation.

## Figures and Tables

**Table 1:** A summary of infant cry dataset.

Group	Hungry	Change Diaper	Emotional Needs	Medical Treatment	Total
Healthy	868	301	486	50	1705
Sick	303	74	425	38	840

**Table 2:** MFCC Parameter.

Parameter	Value
Audio length	2 s
Sampling rate	8000 Hz
Framing	25 ms
Overlapping	10 ms
Number of coefficients	12

**Table 3:** Parameter setting of each classification method.

Method	Parameters
ANN	Activation function: ReLU
	Optimizer: Adam
	Input layer = (201.12)
	Hidden layer 1 = 256 Dropout = 50% Hidden layer 2 = 128 Dropout = 50%
	Output layer = (total class)
	Epoch = 20
CNN	Optimizer Adam
	Input layer = (201.12) Convolutional 1-D = 364, kernel = 3, kernel regulation = l2 (0.01) Max-Pooling 1-D (Kernel = 3) Convolutional 1-D = 180, kernel = 3, kernel regulation = l2 (0.01) Max-Pooling 1-D (Kernel = 3) Global Average Pooling 1-D Hidden layer 1 = 32 Dropout = 40% Output layer = (total class)
	Epoch = 20
LSTM	Input Size = (201.12) Activation function: Sigmoid Optimizer: Adam Number of LSTM Neuron: Hidden layer 1 = 128 Dropout = 5% Recurrent dropout = 35% Return sequences = True Hidden layer 2 = 32 Dropout = 5% Recurrent dropout = 35% Return sequences = False Output layer = (total class) Epoch = 20

**Table 4:** Four-class precision and recall results.

Class	Dataset	Method	Precision				Recall			
			Hungry	Change Diaper	Emotional Needs	Medical Treatment	Hungry	Change Diaper	Emotional Needs	Medical Treatment
Four-class	Full	ANN	0.54	0.06	0.42	0.06	0.51	0.21	0.32	0.01
		CNN	0.57	0.41	0.55	0.07	0.54	0.24	0.54	0.01
		LSTM	0.52	0.22	0.42	0.24	0.55	0.13	0.50	0.03
	Balanced	ANN	0.24	0.40	0.36	0.29	0.27	0.46	0.37	0.22
		CNN	0.54	0.54	0.60	0.53	0.46	0.53	0.59	0.49
		LSTM	0.34	0.35	0.43	0.31	0.36	0.29	0.47	0.35

**Table 5:** Three-class precision and recall results.

Class	Dataset	Method	Precision			Recall		
			Hungry	Change Diaper	Emotional Needs	Hungry	Change Diaper	Emotional Needs
Three-class	Full	ANN	0.51	0.11	0.32	0.37	0.21	0.35
		CNN	0.62	0.50	0.58	0.69	0.12	0.65
		LSTM	0.60	0.27	0.50	0.62	0.12	0.58
	Balanced	ANN	0.42	0.44	0.47	0.44	0.46	0.43
		CNN	0.61	0.55	0.56	0.58	0.58	0.55
		LSTM	0.47	0.44	0.45	0.44	0.45	0.48

**Table 6:** Two-class precision and recall results.

Class	Dataset	Method	Precision		Recall	
			Sick	Healthy	Sick	Healthy
Two-class	Full	ANN	0.96	0.90	0.93	0.93
		CNN	0.96	0.95	0.98	0.89
		LSTM	0.95	0.88	0.94	0.91
	Balanced	ANN	0.90	0.86	0.83	0.89
		CNN	0.94	0.97	0.98	0.94
		LSTM	0.98	0.93	0.93	0.98

**Table 7:** Accuracy of different classes over three methods.

Class	Dataset	ANN	CNN	LSTM
Four-class	Full	0.28	0.55	0.46
	Balanced	0.33	0.64	0.37
Three-class	Full	0.38	0.60	0.54
	Balanced	0.45	0.60	0.45
Two-class	Full	0.92	0.94	0.93
	Balanced	0.87	0.96	0.95

## Data Availability

Available upon request.
